# Quality-assured treatment in certified cancer center networks in upper Franconia, Germany: a population-centered retrospective cohort analysis based on data of the Bavarian cancer registry

**DOI:** 10.1186/s12913-024-11972-3

**Published:** 2024-11-22

**Authors:** Martin Emmert, Ingrid Gorodiscan, Andrea Thater, Doris Buchner, Alexander Kiani, Jacqueline Müller-Nordhorn, Stefan Rohrbacher

**Affiliations:** 1 Bavarian Cancer Registry, Bavarian Food and Health Safety Agency, Bayreuth, Germany; 2 Business & Economics; Quality Management, Health Economics & Preference Research in Oncology, University of Bayreuth, Prieserstraße 2, Bayreuth, 95444 Germany; 3 Klinikum Bayreuth GmbH, Medical Clinic IV, Bayreuth, Germany; 4 Bavarian Cancer Registry, Bavarian Food and Health Safety Agency, Munich, Germany

**Keywords:** Cancer treatment, Certified cancer center, Patient-centered care

## Abstract

**Background:**

Cancer is the second most common cause of death in Germany, and treatment in certified cancer networks is recommended to ensure high-quality care. This study sought to (1) determine the percentage of all primary tumors that might potentially have been treated in certified cancer networks and (2) assess the development and current state of quality-assured cancer care for all cancer patients from a locally defined region in Upper Franconia, Germany.

**Methods:**

This study was a population-centered retrospective cohort analysis based on data from the Bavarian Cancer Registry (2017–2023). First, we determined all potentially available cancer network certifications and calculated the percentage of cancer care that could potentially have been conducted in certified cancer networks. Second, we considered the certification status of the involved healthcare providers and analyzed whether or not cancer care was actually carried out in certified cancer networks.

**Results:**

Overall, 90.1% (62,119/68,973) of all primary tumors, from a total of 63,372 patients, might potentially have been treated in certified cancer networks. The percentage of patients actually receiving care in certified cancer center networks was 40.7% for initial diagnosis, 59.0% for surgery, 53.2% for chemotherapy, and 50.7% for radiotherapy; the weighted mean was 50.3%. The results thus ranged between 46.9% (2023) and 52.8% (2022). The highest proportions of patients who received quality-assured treatment in certified cancer center networks were determined for breast cancer (79.5%), colon cancer (73.1%), and lymphoma (60.1%); in contrast, the lowest results were shown for lung cancer (2.7%), anal cancer (0.0%), and mesothelioma (0.0%). Female patients as well as younger patients were significantly more likely to receive care in certified care networks compared with their counterparts. In addition, we did not find a clear trend whether patients in different tumor stages were more or less likely to receive care in certified care networks.

**Conclusions:**

We found meaningful differences in the proportion of patients who received quality-assured treatment in certified cancer center networks. Following this, patients should receive comprehensive information about receiving care in certified cancer center networks and consider longer travel distances, especially for those cancer types without locally available certified cancer networks.

## Background

Cancer plays a significant role in healthcare practice and is a leading cause of death worldwide. In 2022, almost 10 million people died from cancer worldwide and approximately 20 million people were newly diagnosed with cancer [1]. Oncological diseases are also a very common cause of death in Germany, where cancer is the second most common cause of death after cardiovascular diseases. In 2022, the number of people who died from cancer was reported to be 239,948, accounting for 21.7% of all deaths in Germany [2]. More specifically, lung cancer was the most frequently diagnosed cancer causing death in men, while breast cancer was the most frequent cancer causing death in women [3]. Almost 1.5 million patients were treated for cancer in hospitals in 2021 [4]. Regarding the incidence of cancer, the number of new cases was 493,200 in 2020, with slightly higher numbers in men (261,800) than women (231,400) [5]. In 2020, the costs of illness caused by cancer amounted to almost 44 billion euros, accounting for slightly more than 10% of the total costs of illness (432 billion euros) in Germany [6].

The complexity of the disease, the relatively long and intensive treatment, and the accompanying symptoms require an increased need for interdisciplinary oncological treatment across existing care sectors. The continuous improvement of oncological care structures is of particular relevance to increase the likelihood for a high level of treatment quality [7]. In this context, the German National Cancer Plan from 2008 is considered a milestone for strengthening and further developing the oncological care landscape in Germany [8]. Both the statutory cancer registration in Germany (Cancer Early Detection and Registry Act, § 65c German Social Code Book V) as well as the certification of oncological treatment facilities are therefore of major importance [8]. Regarding the latter, the participation of oncological care facilities in German Cancer Society certification programs requires the fulfilment of entity-specific quality requirements, the implementation of evidence-based treatment guidelines (“S3-guidelines”), among others [8]. In short, “certified cancer centres are tumour-specific networks of inpatient and outpatient facilities in which all medical specialties involved in the treatment of cancer patients work closely together and guarantee continuity of care” [9].

The concept of certified cancer centers is fundamentally based on a three-level certification model [10]. The first level is represented by organ cancer centers, which make up the broad basis to cover cancer care for highly prevalent tumor entities (e.g., breast cancer, colorectal cancer, prostate cancer) [9]. Here, the implementation of organ cancer centers is generally nationwide; to date (March 2023), Germany currently has 1,213 organ cancer centers (1,153 locations) [11]. The second level includes oncology centers that extend to several organs, including non-common cancers. To date (March 2023), Germany currently has 146 oncology centers (155 locations) [11]. At the top of the pyramid are Comprehensive Cancer Centers (CCCs), which have the highest degree of specialization; their focus is particularly on research and teaching in oncology. In Germany, the current 15 CCCs are located directly at university hospitals [12]. (Please note that module and focus options can also be subject to the certification process for certain organs or oncological diseases; see Supplemental Material 2.) With the first certifications of breast cancer centers in 2003 by the German Cancer Society (Deutsche Krebsgesellschaft, DKG), the certification system was implemented in German healthcare practice and has been continuously developed since then [10]. From 2016, cancer centers outside of Germany can be certified as an “European Cancer Centre” through the European Cancer Centre (ECC) Certification Programme [9]. So far, 184 organ cancer centers and 13 oncology centers haven been certified in China, Italy, Luxembourg, Poland, Switzerland, and Austria [13]. To guarantee quality-assured care for certain cancer patients, the patient guidelines of the German Association of Scientific Medical Societies recommend treatment in certified cancer centers (e.g., breast cancer [14]).

So far, evidence has mostly shown clinical benefits for those being treated in certified cancer networks [15–29], while fewer studies have shown contradictory results (e.g., [30, 31]). Nevertheless, an important editorial on the evaluability of the effect of oncology center certification has highlighted some important issues which should be mentioned as well. For example, the author highlighted the still weak evidence base on the importance of center certification compared with other factors, such as hospital case volume or surgeon volume. Also, most studies on the effect of center certification are designed as observational studies which are more susceptible to systematic errors, especially with regard to clinical issues (e.g., selection and information bias, confounding due to unmeasured confounders, poorly measured confounders). Besides this, the impact of a change of the cancer patient’s health care provider might also lead to a certain level of uncertainty [32].

Compared to other diseases, cancer treatment is highly complex and lengthy. The oncological treatment is divided into several sequences (e.g., diagnostics, surgery, chemotherapy, and radiotherapy). Ideally, the course of therapy is tailored to the individual course of the disease and can be ensured or used across several care sectors [7, 33]. Based on the current state of research, however, there is only one pilot study for breast cancer treatment assessing the proportions of care across the individual care sequences being provided in certified and non-certified care networks [34]. This study therefore sought to (1) determine the percentage of all primary tumors that might potentially have been treated in certified cancer networks and (2) assess the development and current state of quality-assured cancer care for all cancer patients from a locally defined region in Upper Franconia, Germany.

In more detail, we sought to address the following four research questions: (1) What percentage of cancer care could potentially have been conducted in certified cancer networks in Upper Franconia, Germany? Are there differences between the different (2) treatment sequences (i.e., diagnostics, surgery, chemotherapy, radiation) and (3) cancer types (i.e., breast cancer, prostate cancer, lung cancer)? (4) Which patients are more likely to receive treatment in certified cancer center networks (e.g., gender, tumor stage, cancer type)? The results might help us learn more about regional quality deficits in cancer care.

## Methodology

This study sought to evaluate the potential and actual level of treatment provided in certified cancer networks in Upper Franconia, Germany, which is one of seven administrative districts in Bavaria, Germany, and covers an area of 7,230 km^2^ with about one million inhabitants [35]. According to data from the Bavarian Cancer Registry, approximately 6,500 people are newly diagnosed with cancer each year [36]. In Upper Franconia, these patients may be treated in 32 local hospitals (number of beds: minimum 12, maximum 967), with six hospitals providing certified cancer centers, although there is no CCC in the region of Upper Franconia. However, it is important to mention that patients in Germany are free to choose any hospital without any regional constraints [37, 38]; following this, patients from Upper Franconia might also choose hospitals outside of the region (see also [39, 40]).

In total, this study comprised five steps. The first two steps are related to the first part of this study, while steps three to five are related to the second part of the analysis. In the first part, we determined all of the available cancer network certification options and calculated the percentage of cancer care which could potentially have been conducted in certified cancer networks (i.e., we evaluated whether there were any certifications available for the different cancer types at the time of treatment). In the second part, we considered the certification status of the involved healthcare providers and analyzed whether or not cancer care was actually carried out in certified cancer networks (see Supplemental Material 1).

First, we provide an overview of certification options according to the German Cancer Society between 2017 and 2023 (see Supplemental Material 2) [13]. We thus determined all possible certificates (e.g., organ cancer centers, oncology centers, modules, focus) for all tumor entities (i.e., organs). For example, the certification “breast cancer center” or “lung cancer center” was available during the entire study period (see above). Based on this, we determined the percentage of cancer care that could potentially have been conducted in certified cancer networks (referred to in this paper as “potential CertCanNet-Patients”) in Upper Franconia in the second step. We thus compared cancer-related diagnosis and treatment data from the Bavarian Cancer Registry with potentially available certification networks. The data set from the Bavarian Cancer Registry contains information on the diagnoses and treatments of cancer patients with a primary residence in Upper Franconia, as well as the healthcare providers involved. For example, 100% of treatment related to breast cancer or lung cancer could potentially have been carried out in certified breast/lung cancer networks, because the corresponding certificates were available during the entire study period.

In the third step, we developed an overview of all hospitals and associated cancer networks (e.g., corresponding physicians from the outpatient sector), including corresponding certifications, in Upper Franconia. All hospitals were thus differentiated according to the main areas of cancer care as well as to certification status [13, 41, 42]. The data were based on public statistics and databases, such as the Bavarian State Office for Statistics and the Bavarian State Ministry of Health and Care [41, 43]. The main part of the search for certified cancer centers and their network partners was based on the OncoMap database of the Institute for Quality Assurance and Data Management in Medicine OnkoZert, which acts as the mandate holder for the German Cancer Society [42]. We also used data from the German Cancer Society [13], surveyed all corresponding hospitals, and used data from the Hospital Quality Reports [44]. It is important to mention that this overview considers all time periods in which each hospital was certificated by any certificate during our study period (4/2017–12/2023). For example, we determined interruptions in the certification of certain tumor types for three hospitals. Based on this, we were able to determine whether or not participating health care providers (e.g., hospitals, outpatient practices) were certified at the moment of the health care delivery.

The fourth step of the analysis was based on a retrospective cohort analysis of healthcare data from the Bavarian Cancer Registry, with a focus on all cancer patients residing in the administrative district of Upper Franconia. Based on our previous steps, we calculated a binary variable (1 = receiving cancer care in certified cancer structures; 0 = not receiving cancer care in certified cancer structures) for each single diagnosis or treatment (i.e., surgery, chemotherapy, radiotherapy). Following this, we analyzed whether or not each diagnosis or treatment was carried out in a certified cancer network. In the fifth step, we determined the percentage of patients receiving cancer care in certified cancer networks by treatment sequences, cancer type (ICD 10 classification), cancer stage, and other characteristics.

In sum, our inclusion criteria referred to patients with any cancer-related diagnosis or treatment information during our study period (4/2017–12/2023). We did not exclude certain subtypes of cancer but included the full range of corresponding ICD 10 codes (C00-D48) since our aim was to provide a comprehensive overview of the full range of cancer-related diagnosis or treatment. However, we excluded patients aged 18 or below since The German Childhood Cancer Registry (GCCR) performs recording the essential data for this group [45]. In addition, we excluded cases with unknown locations of diagnosis or treatment.

Statistical analysis was performed using SPSS (IBM Corp. Released 2019. IBM SPSS Statistics for Windows, Version 26.0. Armonk, NY: IBM Corp) and R Statistical Software (Version 4.2.2; R Foundation for Statistical Computing, Vienna, Austria). Descriptive statistics (i.e., means for continuous variables, percentages for categorical variables) were used to examine demographic and tumor-related variables. Both absolute and relative frequencies, as well as the arithmetic mean and standard deviation, were specified. Case segmentation (e.g., gender, age groups, tumor stage) formed the basis of tabular presentations. In addition, we computed confidence intervals for every level of each category to examine group differences [46, 47]. This approach allows us to compute confidence intervals for multinomial variables and also ensures that the intervals remain in the range of [0,1] even for small observed cell frequencies.

## Results

Overall, the study sample comprised 63,372 patients suffering from 68,973 primary tumors (Table 1). As shown, 90.1% (62,119/68,973) of all primary tumor-related treatments (i.e., diagnosis, treatment) might potentially have been treated in certified cancer networks (potential CertCanNet-Patients). In contrast, 9.9% (6,854/68,973) of all primary tumor-related treatments could not potentially have been treated in certified cancer networks, because there were no certifications available at the time of treatment (non-potential CertCanNet-Patients). Potential CertCanNet-Patients tended to be older (67.4 years vs. 58.8 years), more likely to be male (52.6%; 95% CI: 52.1%−53.1% vs. 31.7%; 95% CI: 30.3%−33.0%), and to suffer from less advanced tumor states. Here, we did not observe an overlap of confidence intervals for any level between both groups. Approximately one third of the registered carcinomas among potential CertCanNet-Patients were diagnosed at stages I (19.4%) or II (12.4%); 4.7% of the tumors had already spread at the time of diagnosis (UICC stage IV); while no information on the UICC stage was available for around half of all tumors (52.2%). Regarding the different treatment sequences, we could detect a documented diagnosis for 47,858 (84.5%) of potential CertCanNet-Patients during the study period. (Please note: the remaining patients were diagnosed before the beginning of our study period, i.e., April 2017, but received treatments afterwards.) In addition, 30,712 (54.2%) of these patients underwent surgery procedures, 23,236 (41.0%) received chemotherapy, and 15,143 (26.7%) were treated with radiotherapy.

**Table 1:** Description of the study sample; patients with or without the potential to be treated in certified cancer networks (CertCanNet-Patients; n = 63,372 patients suffering from 68,973 primary tumors)

	Potential CertCanNet-Patients^§^ (*n* = 56,641)				Non-potential CertCanNet-Patients^&^ (*n* = 6,731)			
	Mean or *n*	SD or %	95% CI		Mean or *n*	SD or %	95% CI	
Gender								
Female	26,866	47.4%	46.9%	47.9%	4,598	68.3%	66.9%	69.7%
Male	29,773	52.6%	52.1%	53.1%	2,132	31.7%	30.3%	33.0%
Missing	2	0.0%	0.0%	0.0%	1	0,0%	0.0%	0.1%
Age*								
Mean^#^ (in years)	67.39	14.11			58.82	19.80		
−50 years	5,580	9.9%	9.5%	10.2%	2,392	35.5%	34.0%	37.1%
50 to 59 years	9,330	16.5%	16.1%	16.9%	873	13.0%	12.0%	14.1%
60 to 69 years	14,886	26.3%	25.8%	26.8%	1,073	15.9%	14.8%	17.1%
70 to 79 years	14,983	26.5%	26.0%	26.9%	1,158	17.2%	16.1%	18.4%
80 + years	11,862	20.9%	20.5%	21.4%	1,235	18.3%	17.2%	19.6%
Place of residence								
City (urban districts)	12,843	22.7%	22.3%	23.1%	1,577	23.4%	22.3%	24.6%
Country (rural districts)	43,798	77.3%	76.9%	77.7%	5,154	76.6%	22.3%	24.6%
Tumor stage								
Stage 0	1,482	2.4%	2.2%	2.6%	117	1.7%	1.3%	2.2%
Stage I	12,050	19.4%	19.0%	19.8%	1,042	15.2%	14.1%	16.4%
Stage II	7,677	12.4%	12.0%	12.7%	1,144	16.7%	15.5%	17.9%
Stage III	5,547	8.9%	8.6%	9.2%	1,060	15.5%	14.3%	16.7%
Stage IV	2,914	4.7%	4.5%	4.9%	881	12.9%	11.8%	14.0%
Missing	32,449	52.2%	51.7%	52.8%	2,610	38.1%	36.5%	39.6%
Number of primary tumors (*n* = 68,973)								
1	51,895	91.6%	91.3%	91.9%	6,613	98.2%	97.8%	98.6%
2	4,228	7.5%	7.2%	7.7%	107	1.6%	1.3%	2.0%
3 +	518	0.9%	0.8%	1.0%	11	0.2%	0.1%	0.3%
Overall	62,119	90.1%	n.a	n.a	6,854	9.9%	n.a	n.a
Treatment sequences (subpopulations)^$^								
Diagnosis	47,858	84.5%	84.1%	84.8%	6,464	96.0%	95.5%	96.5%
Surgery	30,712	54.2%	53.8%	54.7%	3,323	49.4%	48.0%	50.7%
Chemotherapy	23,236	41.0%	40.6%	41.5%	665	9.9%	9.1%	10.7%
Radiotherapy	15,143	26.7%	26.3%	27.2%	404	6.0%	5.4%	6.7%

^§^Patients which might potentially have been treated in certified cancer networks

^&^Patients without the potential to have been treated in certified cancer networks

^*^Date of initial diagnosis

^#^Arithmetic mean

^$^Number and percentage of patients with at least one treatment in each treatment sequence

**Fig. 1:**
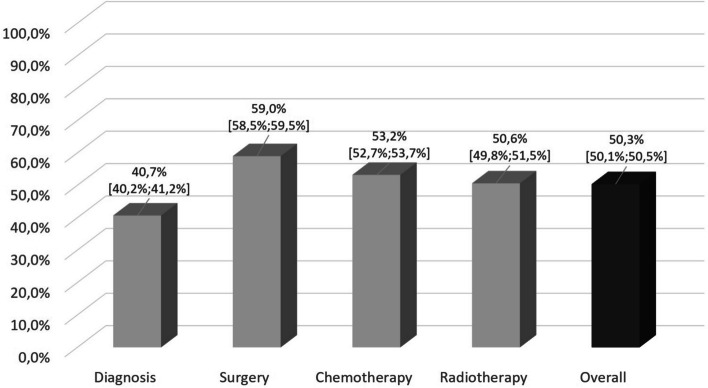
Patients receiving cancer care in certified cancer structures by treatment sequences (in percentage, including 95% CIs) (n = 157,871) in Upper Franconia, Germany between 2017 and 2023

Supplemental Material 3 presents an overview of (non) potential CertCanNet-Patients differentiated by ICD 10 categories. As shown, certification was available for the following ICD 10 categories during the entire study period: C00–C14 (lip, oral cavity and pharynx), C50 (breast), C60–C63 (male genital organs), and C81–C96 (lymphoid, hematopoietic, and related tissue). Based on this, all corresponding cases might thus potentially have been treated in certified cancer networks. Regarding all potential CertCanNet-Patients, we were able to detect 157,871 pieces of diagnosis- or treatment-related information (Fig. 1). Overall, the percentage of patients receiving care in certified cancer center networks was 40.7% (95% CI: 40.2%−41.2%) for initial diagnosis (21,131/51,948), 59.0% (95% CI: 58.5%−59.5%) for surgery (25,081/42,520), 53.2% (95% CI: 52.7%−53.7%) for chemotherapy (23,671/44,475), and 50.6% (95% CI: 49.8%−51.5%) for radiotherapy (9,583/18,928). In sum, slightly more than half (50.3%; 95% CI: 50.1%−50.5%) of all cancer-related treatments (79,466/157,871) were conducted in certified structures.

**Fig. 2:**
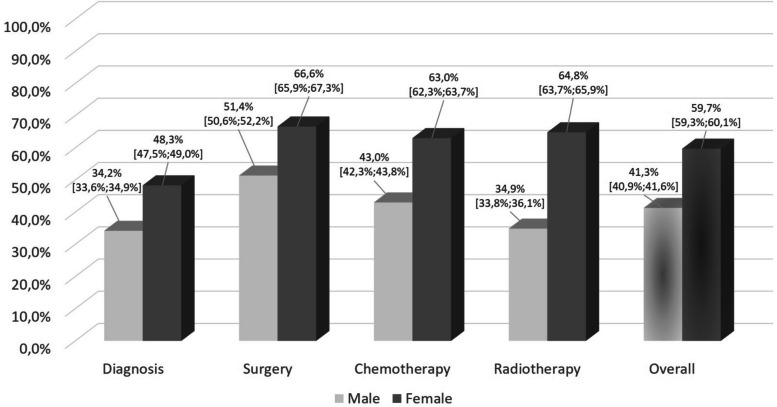
Patients receiving cancer care in certified cancer structures by treatment sequences and gender (in percentage, including 95% CIs) (*n* = 157,871) in Upper Franconia, Germany between 2017 and 2023

Table 2 presents the results regarding cancer care in certified cancer networks by stage and treatment sequences. Here, we did not find a clear trend whether patients in different tumor stages (i.e., I to IV) were more or less likely to receive care in certified care networks. For example, the percentage of patients who received cancer care in certified networks varied between 58.6% (95% CI: 57.8%−59.4%) for stage III and 63.0% (95% CI: 62.2%−63.7%) for stage II, respectively. Please see also Supplemental Material 4 for a comprehensive overview of patients receiving cancer care in certified and non-certified cancer structures. Here, it could be shown that the percentage of patients receiving cancer care in certified care networks decreased with an increasing age; this means that younger patients were significantly more likely to receive care in compared with those in higher age groups. In addition, it could be shown that female patients (59.7%; 95% CI: 59.3%−60.1%) were more likely to receive cancer care in certified care networks compared with male patients (41.3%; 95% CI: 40.9%−41.6%). More in detail, Fig. 2 also shows that female patients were statistically significantly more likely to be treated in certified cancer networks across all therapy sequences (i.e., diagnosis, treatment).

**Table 2:** Patients receiving cancer care in certified cancer networks by stage and treatment sequences (*n* = 157,871) in Upper Franconia, Germany between 2017 and 2023

	Cancer care in certified structures (*n* = 79,466; 50.3%)					Cancer care in non-certified structures (*n* = 78,405; 49.7%)					Overall
	Absolute	In %	95% CI		Absolute		In %	95% CI			
Stage 0											
Diagnosis	881	73.8%	70.9%	76.6%	312		26.2%	23.4%	29.1%	1,193	
Surgery	1,399	77.7%	75.4%	79.8%	401		22.3%	20.2%	24.6%	1,800	
Chemotherapy	729	77.9%	74.7%	80.8%	207		22.1%	19.2%	25.3%	936	
Radiotherapy	510	77.7%	73.9%	81.2%	146		22.3%	18.8%	26.1%	656	
Overall	3,519	76.8%	75.3%	78.1%	1,066		23.2%	21.9%	24.7%	4,585	
Stage I											
Diagnosis	5,203	49.7%	48.6%	50.8%	5,263		50.3%	49.2%	51.4%	10,466	
Surgery	7,945	60.1%	59.2%	61.1%	5,267		39.9%	38.9%	40.8%	13,212	
Chemotherapy	2,944	70.3%	68.7%	71.8%	1,245		29.7%	28.2%	31.3%	4,189	
Radiotherapy	2,020	70.0%	68.0%	71.9%	866		30.0%	28.1%	32.0%	2,886	
Overall	18,112	58.9%	58.3%	59.5%	12,641		41.1%	40.5%	41.7%	30,753	
Stage II											
Diagnosis	3,304	54.7%	53.3%	56.1%	2,735		45.3%	43.9%	46.7%	6,039	
Surgery	5,150	65.2%	64.0%	66.4%	2,750		34.8%	33.6%	36.0%	7,900	
Chemotherapy	3,264	65.8%	64.3%	67.3%	1,697		34.2%	32.7%	35.7%	4,961	
Radiotherapy	1,941	69.5%	67.5%	71.4%	853		30.5%	28.6%	32.5%	2,794	
Overall	13,659	63.0%	62.2%	63.7%	8,035		37.0%	36.3%	37.8%	21,694	
Stage III											
Diagnosis	2,090	49.0%	47.3%	50.8%	2,172		51.0%	49.2%	52.7%	4,262	
Surgery	3,758	61.6%	60.2%	63.0%	2,345		38.4%	37.0%	39.8%	6,103	
Chemotherapy	3,266	62.0%	60.5%	63.5%	2,001		38.0%	36.5%	39.5%	5,267	
Radiotherapy	1,505	60.5%	58.3%	62.7%	981		39.5%	37.3%	41.7%	2,486	
Overall	10,619	58.6%	57.8%	59.4%	7,499		41.4%	40.6%	42.2%	18,118	
Stage IV											
Diagnosis	1,160	52.8%	50.4%	55.1%	1,039		47.2%	44.9%	49.6%	2,199	
Surgery	2,198	65.7%	63.8%	67.5%	1,148		34.3%	32.5%	36.2%	3,346	
Chemotherapy	2,503	63.5%	61.8%	65.2%	1,438		36.5%	34.8%	38.2%	3,940	
Radiotherapy	826	55.1%	52.2%	57.9%	674		44.9%	42.1%	47.8%	1,500	
Overall	6,687	60.9%	59.8%	61.9%	4,299		39.1%	38.1%	40.2%	10,985	
Missing stage											
Diagnosis	8,494	30.6%	30.0%	31.2%	19,291		69.4%	68.8%	70.0%	27,785	
Surgery	4,631	45.7%	44.6%	46.8%	5,507		54.3%	53.2%	55.4%	10,138	
Chemotherapy	10,966	43.5%	42.8%	44.2%	14,227		56.5%	55.8%	57.2%	25,193	
Radiotherapy	2,782	32.3%	31.2%	33.5%	5,818		67.7%	66.5%	68.8%	8,600	
Overall	26,873	37.5%	37.1%	37.9%	44,843		62.5%	62.1%	62.9%	71,716

**Fig. 3:**
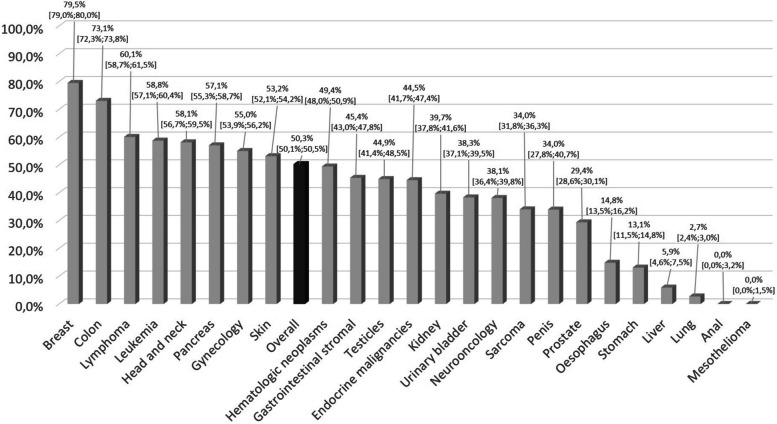
Patients receiving cancer care (i.e., diagnosis, therapy) in certified cancer structures by cancer type (in percentage, including 95% CIs) (n = 157,871) in Upper Franconia, Germany between 2017 and 2023

Finally, the highest proportion of patients who received quality-assured treatment in certified cancer center networks in Upper Franconia, Germany, between 2017 and 2023 was determined for breast cancer (79.5%; 95% CI: 79.0%−80.0%), colon cancer (73.1%; 95% CI: 72.3%−73.8%), lymphoma (60.1%; 95% CI: 58.7%−61.5%), and leukemia (58.8%; 95% CI: 57.1%−60.4%) (Fig. 3); in contrast, the lowest results were shown for liver cancer (5.9%; 95% CI: 4.6%−7.5%), lung cancer (2.7%; 95% CI: 2.4%−3.0%), anal cancer (0.0%; 95% CI: 0.0%−3.2%), and mesothelioma (0.0%; 95% CI: 0.0%−1.5%) (Supplemental Material 5 presents the results aggregated by ICD 10 classification). Regarding developments over time (Supplemental Material 6), the results ranged between 46.9% (2023) and 52.8% (2022) of patients receiving quality-assured treatment in certified cancer center networks, but no clear trend was discernible.

## Discussion

The aim of this study was to (1) determine the percentage of all primary tumors which might potentially have been treated in certified cancer networks and (2) assess the development and current state of quality-assured cancer care for all cancer patients from a locally defined region in the administrative district of Upper Franconia, Germany, between 2017 and 2023. We therefore examined the proportion of patients who could potentially have been treated in certified cancer networks and, among those, how many were actually treated in certified cancer networks during the individual treatment sequences (i.e., diagnosis, surgery, chemotherapy, and radiotherapy).

First, it is important to mention that our study assumes, at least to some extent, that cancer care in certified cancer networks shows favorable results compared to care in non-certified cancer networks. As mentioned above, the evidence so far predominantly shows more favorable outcomes if cancer care is conducted in certified cancer networks [16, 19–22, 48–51]. For example, the recently published German WiZen study (Effectiveness of Care in Certified Cancer Centers) [48, 52] showed consistently longer overall survival times for patients who had received initial treatment in a certified cancer center for all entities studied [52]. This result is in line with previous evidence from other studies demonstrating a favorable outcome (i.e., overall survival) for patients in certified breast cancer centers [19, 20, 50], pancreatic cancer centers [21], colon and rectal cancer centers [22], or other certified cancer-related centers (see above) in Germany. One reason for the predominantly positive effect of certified cancer centers can be assumed to be that the quality specifications in certified facilities lead to improvements in structural and process quality, which are linked to guideline-based and entity-specific quality indicators and thus have a positive effect on patient care [21, 49]. However, it should be mentioned that there have also been studies that could not prove the positive impact of certifications on quality of care. For example, a study by Schrodi [30] concluded that certification did not have an effect on the survival of younger breast cancer patients. In addition, a systematic review that analyzed the effects of treatments in certified centers did not draw any clear conclusions regarding patient care or treatment effectiveness [31]. As stated above, a recent editorial has also highlighted some factors which should be considered when analyzing the effect of oncology center certification. Here, the author highlighted the still weak evidence base on the center or certification effect compared with other factors, such as hospital case volume or surgeon volume. Also, most studies on the effect of center certification (e.g., such as the above mentioned WiZen study [48, 52]) are designed as observational studies which are more susceptible to systematic errors, especially with regard to clinical issues (e.g., selection and information bias, confounding due to unmeasured confounders, poorly measured confounders). Finally, the impact of a change of the cancer patient’s health care provider might also lead to a certain level of uncertainty [32]. The literature has also mentioned the high financial costs resulting from tasks required for the certification of a certified cancer center [29]. For example, one study estimated the costs for a Comprehensive Cancer Center (CCC) totaled up to €4.9 million and those for an organ cancer center to reach approximately €0.2 million per year (in 2016 Euros) [53].

Second, we showed that 90% of all primary tumor-related treatments might potentially have been treated in certified cancer networks. In contrast, 10% could not potentially have been treated in certified cancer networks due to the non-availability of the necessary certificates at the moment of diagnosis or treatment. This means that a large proportion of patients could theoretically have been treated in quality-assured networks; this demonstrates the availability of certificates for the most important tumor entities in terms of quantity. It can also be seen that most cancer cases without theoretically available certified care networks are related to early stages of cancer. For example, most of those cases can be assigned to the IDC group D00-D09 (in situ neoplasms; 4,232/6,854 or 62%) representing a “malignant epithelial neoplasm which is confined to the epithelial layer without evidence of further tissue invasion” [54]. In case, we would only consider malignant neoplasms (i.e., C00-C97), the percentage of all primary tumor-related treatments which might potentially have been treated in certified cancer networks would increase to 96.3% (57,290/59,522).

Nevertheless, it should be noted that the availability of certificates should not be equated with the ability to fulfill all requirements of individual certificates from a regional perspective. For example, to be certified as a lung cancer center, the center must—besides other requirements—treat at least 200 patients a year with a primary diagnosis of “lung cancer” (ICD, C34.0–34.9) [55]. According to data from the Bavarian Cancer Registry, the total number of lung cancer cases in Upper Franconia varied between 600 cases in 2019 and 687 cases in 2016 [56]; the number of cases treated in the five largest hospitals in Upper Franconia varied from 71 to 165 cases in 2021 and from 56 to 162 cases in 2022. Therefore, despite theoretical availability in a rural region such as Upper Franconia, it seems very challenging to establish a corresponding lung cancer center; it should be mentioned that there is still no lung cancer center in Upper Franconia due to the high requirements. Theoretically, this could only be achieved through appropriate cooperation agreements, if the requirements allow for those, or by actively guiding patients to the relevant centers. As stated previously, this might have a high potential to improve outcomes [51].

Third, we were able to demonstrate that slightly more than half of all cases that might potentially have been treated in certified cancer networks were actually conducted in certified structures (50.3%). This result is slightly higher than was found in previous studies, which showed that more than 40% of all cancer patients did not receive initial cancer treatment in certified cancer center networks in Germany [52]. However, it has to be mentioned that our study focused on the time period between 2017 and 2023, while the WiZen study observed the time period between 2009 and 2017; this may explain (at least to some extent) the differences in the findings. Furthermore, we also included all tumor entities and thus extended the WiZen study, which focused on 11 cancer entities (e.g., colon, rectum, pancreas). Another difference can be found in the study design; while our study relates to the initial diagnosis and three treatment sequences (i.e., surgery, chemotherapy, and radiotherapy), the WiZen study calculated their findings based on whether the initial treatment of patients was conducted in a certified cancer center [51, 52]. Our results therefore provide even more detailed insights into oncological healthcare delivery and go beyond those of the WiZen study. We can show, for example, that the percentage of patients receiving care in certified cancer center networks was 40.7% for initial diagnosis, 59.0% for surgery, 53.2% for chemotherapy, and 50.6% for radiotherapy. In contrast to the WiZen study, we did not detect an increase over time in the proportion of patients treated in certified cancer centers [37]; our results ranged between 46.9% (2023) and 52.8% (2022), without showing a clear trend.

Next, we saw relatively large differences in the proportion of patients who received quality-assured treatment in certified cancer center networks between single tumor entities, which confirms previous findings [22]. For example, the highest proportion was determined for breast cancer (79.5%), colon cancer (73.1%), and lymphoma (60.1%), while the lowest results were shown for liver cancer (5.9%), lung cancer (2.7%), anal cancer (0.0%), and mesothelioma (0.0%). This is in line with previous evidence showing both the highest and similar results in the proportion of patients treated in certified cancer center networks for breast cancer (70% [52], 87% [57], and 83% [58]). Studies have also shown lower results for colorectal cancer patients (e.g., 47% [59], 53% [52], 33% [26]) and similar results for skin cancer (59%) and gynecological tumors (51%) [57]. In addition, Werthemann and Weißbach [57] as well as Rückher and colleagues [58] demonstrated a relatively large spread in the proportion of cancer cases treated in certified cancer centers across individual tumor entities.

Nevertheless, we could also see that cancer care in Upper Franconia indeed shows potential for improvement for very prevalent entities; for example, only 2.7% of lung cancer patients from Upper Franconia were treated in certified cancer center networks. This is lower than in the WiZen study, where approximately 30% of all lung cancer patients were initially treated in certified hospitals and is lower than in another study that showed a proportion of around 24% of lung cancer patients treated in a certified lung cancer network [60]. However, the low result for Upper Franconia here is not surprising, because both the total number of lung cancer patients and the distribution of these patients across hospitals make it very challenging to establish a certified lung cancer network (see above). The data from the Bavarian Cancer Registry show that almost all lung cancer patients who were treated in a certified lung cancer network travelled around 100 km to reach the nearest certified center, which is located in Nuremberg, Middle Franconia.

Finally, we found that female patients were statistically significant more likely to be treated in certified cancer networks across all diagnosis and treatment-related sequences. For example, 63.0% of all female patients received chemotherapy in certified cancer networks, but only 43.0% of all male patients did so (p < 0.001). To a certain extent, this finding is not surprising, because gender-focused certified cancer networks in Upper Franconia are more likely to be related to the treatment of female tumors (e.g., breast cancer, gynecologic cancers) than to the treatment of male tumors (e.g., prostate cancer). For example, six certified breast cancer networks had been established over the entire study period in Upper Franconia, but only two certified prostate cancer networks. In addition, we determined that older patients were less likely to receive cancer care in certified structures. In this regard, we could see a constant decrease in the percentage of patients who received care in certified cancer networks with an increasing age. This finding is mostly in line with other studies which have also demonstrated that patients receiving cancer treatment in certified structures could be shown to be younger than patients receiving cancer treatment in non-certified structures [19, 24, 30, 61]; for example, Beckmann and colleagues showed that patients receiving treatment in certified breast cancer structures were on average two years younger (60.6 vs. 62.9 years; p < 0.00001) [19]. However, it should also be mentioned that other studies did not find meaningful age-related differences between both patient groups [21, 22, 26, 29].

Our findings should be considered in light of some limitations. First, it is important to mention that this study was conducted in Germany and might be of limited relevance for other countries. Nevertheless, the results presented in this paper are of interest for all countries with similar certification programs. As mentioned above, the certification program of the German Cancer Society has been applied outside of Germany from 2016 by means of the ECC Certification Programme (e.g., Italy, Luxembourg, Poland, Switzerland, Austria) [9, 13]. It should be mentioned that research from other countries has also explored the impact of cancer accreditation on outcomes (e.g., survival). For example, a recent study from the United States has shown that the Commission on Cancer (CoC) accreditation had a significant impact on survival in 5 of 59 solid organ cancers [61]. It is important to mention, that the Commission on Cancer (CoC) accreditation may differ in some aspects from the ECC Certification Programme. Nevertheless, both initiatives aim to set comprehensive standards for cancer care; for example, accreditation by the CoC means that patients receive comprehensive, patient-centered care through a multidisciplinary team-approach, access to information on clinical and new treatment options, ongoing monitoring of care, psychosocial support, as well as continuous quality improvements in care [62]. Thus, even though our study focused on data from the German setting, it might also be of interest for other countries. Second, the research design of the present study is based on secondary data analysis. Due to the retrospective approach of this study, missing and erroneous data in the data set could not be excluded. For example, the UICC stage was only available for around half of all tumors (52.2%); this is especially true regarding non-certified structures (here, the percentage of missing UICC stage information was determined to be 57.2%). Following this, the corresponding results should be interpreted with caution. Basically, the registration process for incoming reports to cancer registries is accompanied by various sources of error that can occur at different points in time. These include spelling and typing errors in the processing and documentation of reports received by the cancer registry or errors in the content when filling out the report form [7]. The extent of these effects is compensated for by, among other things, the high demands on quality assurance within the framework of German cancer registration. Nevertheless, it should also mentioned that our analysis is characterized by detailed information on the entire course of diagnosis and therapy that allowed differentiation between surgery, chemotherapy, and radiation. It can also be assumed that the described care situation in Upper Franconia is very likely to provide a realistic picture, as the database is characterized by a high degree of completeness due to the legally anchored reporting system (between 2017 and 2021, the completeness of the reports in Upper Franconia varied between 92 and 100%) [63]).

## Conclusions for health policy makers

In sum, our results show that 90% of cancer patients in Upper Franconia, Germany, could potentially be treated in certified cancer networks. However, the availability of specific certificates should not be equated with the ability to fulfill all the requirements of the individual certificates from a regional perspective; the total number of patients, as well as the distribution of patients across hospitals in a region, can make it unlikely that certain certifications might be realistic. Slightly more than half of all cases (50.3%) that might potentially have been treated in certified cancer networks were actually treated in certified structures. Although the results of the present study appear positive at first glance, they also show an enormous need for action. Overall, the predominantly convincing results for more favorable outcomes if cancer care is provided in certified cancer networks care [16, 19–22, 48–51], as well as the National Cancer Plan in Germany, make it seem necessary to further increase the proportion of patients receiving high quality-assured care. More specifically, it seems necessary to narrow the gap regarding the proportion of patients who receive treatment in certified cancer networks across single tumor entities. Possible regional cooperation agreements or actively guiding patients to relevant networks—even those associated with longer travel distances—might have the potential to improve outcomes. It should at least be assured that patients receive comprehensive information about possible certified cancer networks and their specific advantages so they can make an informed choice.

## Data Availability

The data that support the findings of this study are available on request from the corresponding author [ME]. The data are not publicly available due to privacy restrictions.

## Abbreviations

CCC: Comprehensive Cancer Center
DKG: Deutsche Krebsgesellschaft (German Cancer Society)
ECC: European Cancer Centre
CertCanNet: Certified cancer network
ICD: International Statistical Classification of Diseases and Related Health Problems
UICC: Union Internationale Contre le Cancer
